# Quantitative Difference in Solubility of Diastereomeric (^2^H/^1^H)-Isotopomers

**DOI:** 10.1021/jacs.1c09253

**Published:** 2021-11-05

**Authors:** Tsuneomi Kawasaki, Hiroki Kubo, Satoshi Nishiyama, Taiki Saijo, Rintaro Yokoi, Yuji Tokunaga

**Affiliations:** † Department of Applied Chemistry, Tokyo University of Science, Kagurazaka, Shinjuku-ku, Tokyo 162-8601, Japan; ‡ Department of Materials Science and Engineering, University of Fukui, Bunkyo, Fukui 910-8507, Japan

## Abstract

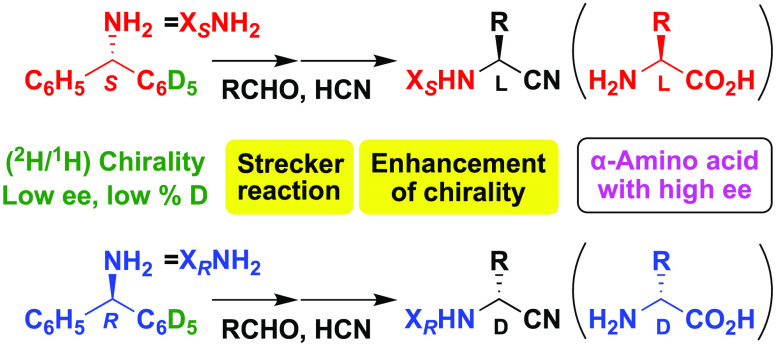

Many achiral organic compounds become chiral by an isotopic substitution of one of the enantiotopic moieties in their structures. Although spectroscopic methods can recognize the molecular chirality due to an isotopic substitution, the effects of isotopically chiral compounds in enantioselective reactions have remained unsolved because the small chirality arises only from the difference between the number of neutrons in the atomic nuclei. The difference between the diastereomeric isotopomers of reactive sources should be the key to these effects. However, the energy difference between them is difficult to calculate, even using present computational methods, and differences in physical properties have not yet been reported. Here, we demonstrate that the small energy difference between the diastereomeric isotopomers at the molecular level can be enhanced to appear as a solubility difference between the diastereomeric (^2^H/^1^H) isotopomers of α-aminonitriles, synthesized from an isotopically chiral amine, achiral aldehyde, and HCN. This small, but measurable, difference induces the chiral (d/l) imbalance in the suspended α-aminonitrile; therefore, a second enhancement in the solid-state chirality proceeds to afford a highly stereoimproved aminonitrile (>99% selectivity) whose handedness arises completely from the excess enantiomer of isotopically chiral amine, even in a low enantiomeric excess and low deuterium-labeling ratio. Because α-aminonitriles can be hydrolyzed to chiral α-amino acids with the removal of an isotope-labeling moiety, the current sequence of reactions represents a highly enantioselective Strecker amino acid synthesis induced by the chiral hydrogen (^2^H/^1^H) isotopomer. Thus, hydrogen isotopic chirality links directly with the homochirality of α-amino acids via a double enhancement of α-aminonitrile, the chiral intermediate of a proposed prebiotic mechanism.

## Introduction

Since Pasteur discovered molecular asymmetry,^1^ the origin of biological chirality in nature has been an attractive mystery.^2,3^ Among the theories,^4−9^ isotopic chirality is a possibility^10^ because many apparently achiral organic compounds become chiral when taking isotopic substitutions into consideration.^11−14^ A higher deuterium-labeling ratio of meteoritic compounds than found in the same terrestrial compounds has been reported;^15^ therefore, extra-terrestrial organic compounds may have isotopic chirality. Although chiral recognition technologies have been developed^16,17^ and isotopically labeled compounds are often used to elucidate reaction mechanisms in the field of stereochemistry and biochemistry, chiral induction effects in asymmetric reactions are difficult to observe. For example, there are reports of kinetic resolutions^18^ or enantioselective reactions^19^ induced by chiral compounds arising from hydrogen isotope (^2^H/^1^H) substitutions; however, the optical yields remain very low, which can be classified as cryptochirality.^20^ Moreover, attempts at the optical resolution of racemic chiral isotopomers by forming diastereomeric salts has been reported.^21^ When labeled and unlabeled compounds are compared not for the diastereomeric isotopomers, structural steric isotope effects^22^ and conformational kinetic isotope effects^23^ have apparently demonstrated that the steric requirement of the C–D bond is smaller than that of the C–H bond.^24^ In a reaction including an asymmetric amplification,^25^ isotope chirality (^2^H/^1^H) has been shown to control macromolecular helical handedness cooperatively,^26^ and supramolecular helical aggregates have been synthesized^27^ from isotopically chiral monomers. In addition, the asymmetric autocatalysis (Soai reaction)^3,28,29^ can be triggered by highly enantioenriched chiral compounds arising from deuterium substitutions such as benzyl alcohol-α-*d*^30^ and glycine-α-*d*.^14^ Recently, attrition-enhanced deracemization has been reported under the competition of labeled and unlabeled enantiomorphs.^31^

We reported^32^ a spontaneous absolute asymmetric Strecker-type synthesis based on the crystallization of chiral intermediate α-aminonitriles.^33−35^ Thus, after the total spontaneous resolution, a small imbalance between enantiomorphs of α-aminonitrile with as low as ca. 0.05% enantiomeric excess (ee) could be significantly amplified to afford a nearly enantiomerically pure solid product by the repetition of a partial dissolution and crystallization cycle under a solution-phase racemization.^36^ Thus, in the present research, by introducing a hydrogen isotope chirality to an achiral starting substrate, a stereoselective synthesis induced by deuterium substitutions was achieved to correlate the hydrogen isotope chirality and chiral l- and d-α-amino acids with high ee via the suggested prebiotic Strecker-type mechanism. The present amplification of solid-state chirality originated from the small but measurable solubility difference between the isotopomers. It is the first example to demonstrate quantitatively a solubility difference between the diastereomeric (^2^H/^1^H) isotopomers and their asymmetric amplification. These effects may be the key to understand the asymmetric induction by enantioenriched chiral compounds arising from hydrogen isotope substitution.

## Results and Discussion

**Figure 1:**
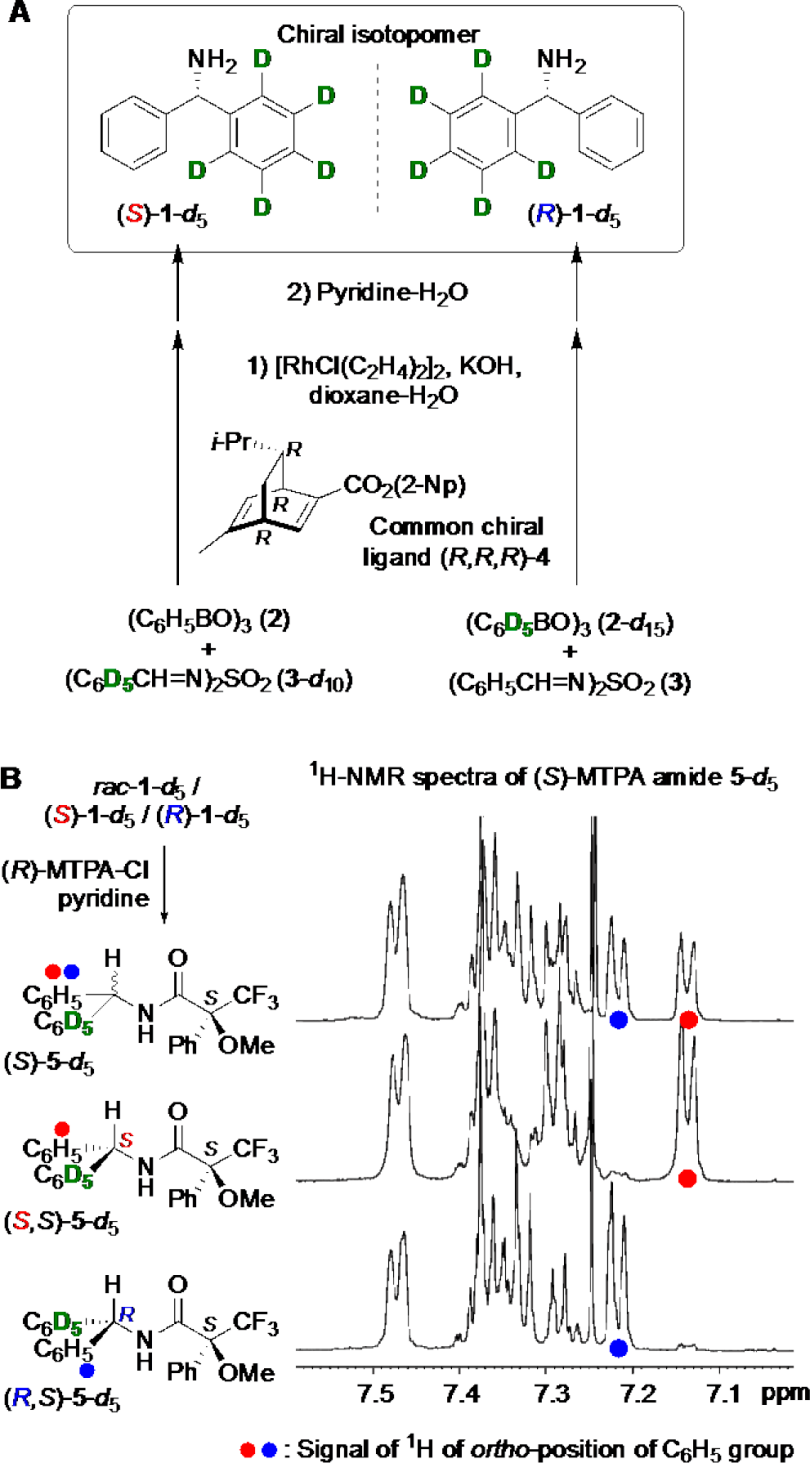
Asymmetric synthesis and evaluation of the enantiopurity of (*S*)- and (*R*)-benzhydrylamine-*d*_5_ (**1**-*d*_5_). (A) Catalytic asymmetric synthesis of (*S*)- and (*R*)-benzhydrylamine-*d*_5_ (**1**-*d*_5_) using the common chiral source (*R,R,R*)-**4**. (B) Determination of enantiopurities of asymmetrically synthesized (*S*)- and (*R*)-**1**-*d*_5_ by ^1^H NMR of their MTPA amides **5**-*d*_5_, respectively.

Benzhydrylamine (**1**) is an achiral primary amine; however, it becomes chiral by a deuterium substitution of one of the two enantiotopic phenyl groups, whose enantiomers (*S*)- and (*R*)-**1**-*d*_5_ are synthesized by the rhodium-catalyzed asymmetric addition of triphenylboroxine **2** to bis-sulfamyl imine **3** using (*R,R,R*)-**4**^37^ as a common chiral ligand (Figure 1A). When unlabeled phenylboroxine **2** was reacted with labeled *N,N*′-bis(benzylidene)sulfamide (**3**-*d*_10_) in the presence of (*R,R,R*)-**4**, (*S*)-**1**-*d*_5_ was obtained after the hydrolysis of the coupling product. By contrast, when unlabeled **3** was treated with labeled boroxine **2**-*d*_15_ by using the same chiral ligand (*R,R,R*)-**4**, oppositely configured (*R*)-**1**-*d*_5_ could be synthesized. The ee values of **1**-*d*_5_ could be determined by derivatizing it to a diastereomeric isotopomer of (*S*)-methoxytrifluoromethylphenylacetamide (MTPA amide) **5**-*d*_5_ (Figure 1B); thus, a ^1^H NMR analysis of **5**-*d*_5_ confirmed amine **1**-*d*_5_ to be ca. 90% ee. A chemical shift difference was observed between the diastereotopic ortho protons of unlabeled phenyl groups.

**Figure 2:**
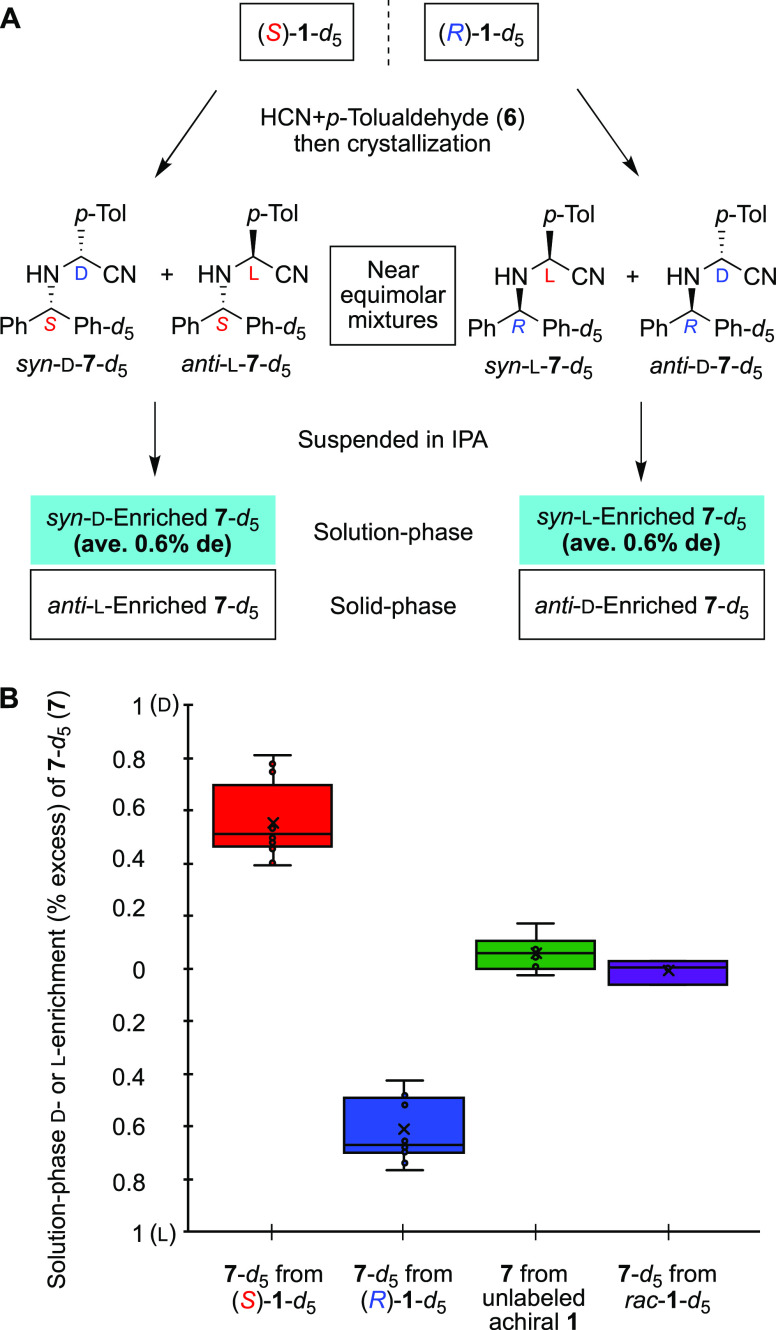
Strecker synthesis of α-aminonitrile **7**-*d*_5_ and the solubility difference between diastereomeric isotopomers of *syn-* and *anti*-**7**-*d*_5_ synthesized from **1**-*d*_5_. (A) Schematic outline of the formation of a diastereomerically imbalanced suspension of aminonitrile **7**-*d*_5_ induced by a hydrogen isotope chirality. (B) Box and whisker plots of a diastereomeric (enantiomeric) excess of the solution-phase aminonitriles **7**-*d*_5_ and **7** in their suspension originating from (*S*)-, (*R*)-, *rac*-**1**-*d*_5_, and unlabeled **1**.

Next, the synthesized (*S*)-**1**-*d*_5_ was subjected to the Strecker reaction between achiral HCN and *p*-tolualdehyde (**6**) (Figure 2A). Thus, after the formation of the corresponding imine, the HCN addition proceeded to give aminonitrile **7**-*d*_5_ as a diastereomeric mixture of a nearly equimolar amount of *syn*-d- and *anti*-l-**7**-*d*_5_. In the case of cryptochirality,^20^ any detectable selectivity was not observed under the homogeneous reaction. As reported previously, unlabeled l- and d-aminonitrile **7** form a conglomerate; therefore, diastereomeric isotopomers *syn*-d-**7**-*d*_5_ and *anti*-l-**7**-*d*_5_, necessarily acting like enantiomers, crystallize to form a separate crystalline solid.

The resulting mixture was suspended in 2-propanol, and the supernatant that was saturated with both diastereomeric isotopomers **7**-*d*_5_ was analyzed using chiral high-performance liquid chromatography (HPLC). Because chiral HPLC could not recognize the stereogenic center arising from deuterium substitution,^38^ only the ratio of d- and l-handedness of **7**-*d*_5_ is analyzable, discriminating usual (nonisotopic) stereogenic centers. Neglecting the isotope chirality, ∼0.6% ee (d-enrichment) was observed as the average value of 12 measurements (Figure 2B and Table S1). Therefore, diastereomeric isotopomers *syn*-d-**7**-*d*_5_ and *anti*-l-**7**-*d*_5_ show a small solubility difference, with *syn*-d-**7**-*d*_5_ being more soluble than *anti*-l-**7**-*d*_5_ in 2-propanol.

By contrast, by utilizing the mixture of diastereomeric aminonitriles **7**-*d*_5_ synthesized from oppositely configured (*R*)-amine-**1**-*d*_5_, near-symmetrical results were obtained; that is, chiral HPLC indicated ca. 0.6% enrichment of *syn*-l-isomer compared with *anti*-d-isomer. Because 13.0 mg of a diastereomeric mixture of aminonitriles **7**-*d*_5_ can be dissolved in 1.0 mL of 2-propanol in their suspension, the solubilities of *syn*- and *anti*-**7**-*d*_5_ were calculated to be 6.54 and 6.46 g/L, respectively. Therefore, in the solid phase, the enrichment of *anti*-l- (from (*S*)-**1**-*d*_5_) and *anti*-d-aminonitrile **7**-*d*_5_ (from (*R*)-**1**-*d*_5_) should be induced in these suspensions, respectively. The control experiments using unlabeled achiral amine **1** and *rac*-amine **1**-*d*_5_ indicated that the observed ee (isotopic diastereomeric excess (de)) values are above the level detectable by current HPLC measurements. Therefore, the solubility imbalances between the *syn*- and *anti*-diastereomeric isotopomers of aminonitriles **7**-*d*_5_ could be observed quantitatively by the chiral HPLC analyses.

To confirm the solubility difference between the diastereomers of aminonitrile **7**-*d*_5_, an improvement of solid-state chirality^39,40^ was conducted (Table 1) based on our previous reports,^36^ in which unlabeled enantiomeric l- and d-**7** with as low as ca. 0.05% ee could be successfully amplified to be greater than 99.5% ee in the solid state under a solution-phase racemization. We proposed that the method could enhance the diastereomeric imbalance of *anti*- and *syn*-isotopomers **7**-*d*_5_. Therefore, aminonitrile **7**-*d*_5_ was submitted to an amplification cycle, in which apparently 80–90% of suspended solid **7**-*d*_5_ was dissolved by heating, then cooling, to induce its recrystallization from the epimerizing solution. After a repetition of this cycle, the resulting solid was isolated by filtration, and we analyzed the ratio of l- and d-**7**-*d*_5_.

**Table 1:**
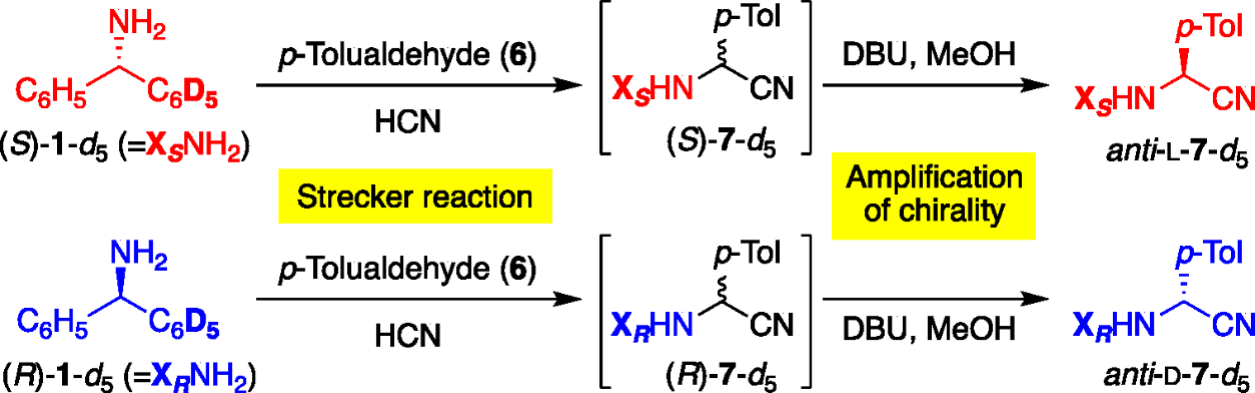
Stereochemical Relationships between the Chiral Isotopomer Benzhydrylamine-*d*_5_ (1-*d*_5_) and Resulting α-Aminonitrile 7-*d*_5_ (Including Unlabeled 7)^a^

			aminonitrile **7**-*d*_5_ (including unlabeled **7**)	
run	configuration of amine **1**-*d*_5_ (% ee) [reaction batch number]^b^	unlabeled **1** (%)	configuration^c^ (% ee)	yield^d^ (%)
1	*S* (91) [#06_*S*_]		l (>99)	56 (23)
2	*R* (83) [#07_*R*_]		d (>99)	56 (25)
3	*S* (89) [#08_*S*_]		l (98)	46
4	*R* (89) [#09_*R*_]		d (99)	47
5	*S* (65) [mixture of #06_*S*_ and #07_*R*_]		l (>99)	56
6	*S* (45) [mixture of #10_*S*_ and #11_*rac*_]		l (98)	63
7	*S* (24) [mixture of #06_*S*_ and #07_*R*_]		l (>99)	51
8	*R* (67) [mixture of #06_*S*_ and #07_*R*_]		d (89)	56
9	*R* (48) [mixture of #04_*R*_ and #11_*rac*_]		d (99)	48
10	*R* (20) [mixture of #06_*S*_ and #07_*R*_]		d (>99)	59
11	*S* (90) [#10_*S*_]	50	l (91)	27
12	*S* (90) [#10_*S*_]	80	l (99)	36
13	*R* (93) [#04_*R*_]	50	d (96)	44
14	*R* (93) [#04_*R*_]	80	d (99)	35

a The molar ratio of **1**-*d*_5_ (+ unlabeled **1**)/aldehyde **6** = 1:1, and an excess amount of HCN was used.

b Identification of **1**-*d*_5_ synthesized from different reaction batch. Labeled amine **1**-*d*_5_ with a low ee was prepared by mixing enantioenriched (*S*)-**1**-*d*_5_ with (*R*)-**1**-*d*_5_ or enantioenriched (*S*/*R*)-**1**-*d*_5_ with *rac*-**1**-*d*_5_ (reaction batch number #11_*rac*_), which was prepared from benzonitrile and PhMgBr-*d*_5_ and following a one-pot LiAlH_4_ reduction of the resulting iminium salt.

c An HPLC analysis using a chiral stationary phase cannot discriminate the isotopic chiral carbon center. Therefore, the ratio of *anti*-l- and *syn*-l-**7**-*d*_5_ the same as *anti*-d- and *syn*-d-**7**-*d*_5_ could not be determined, and the value observed was described as the ee of l- and d-aminonitrile **7**-*d*_5_ and **7**.

d The chemical yield of solid **7**-*d*_5_ by the filtration. The recovered yield of **7**-*d*_5_ from the filtrate is indicated in parentheses.

**Figure 3:**
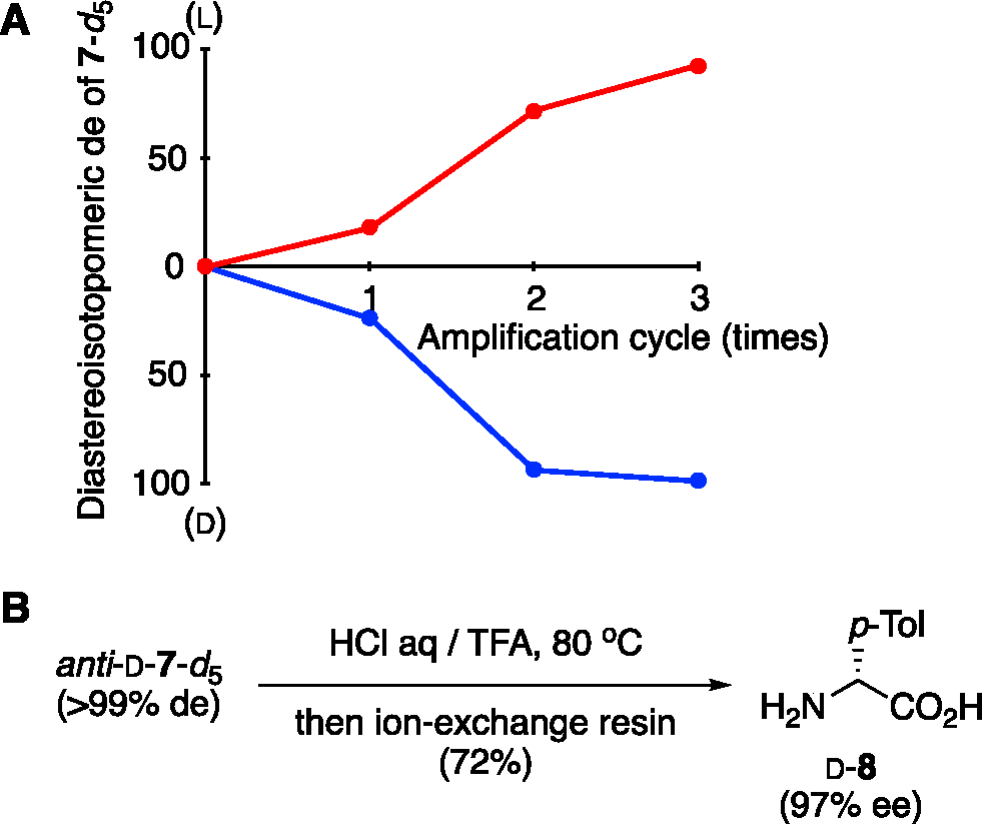
Enantioselective synthesis of α-(*p*-tolyl)glycine (**8**) with enantioenriched isotopically (^2^H/^1^H) chiral benzhydrylamine-*d*_5_ (**1**-*d*_5_) as a source of chirality. (A) Amplification of chirality of α-aminonitrile **7**-*d*_5_ by the heating–cooling cycle. The changes in de of the reactions shown in Table 1, entries 3 and 4, were monitored and were described as red and blue lines, respectively. (B) Acidic hydrolysis of *anti*-d-aminonitrile **7**-*d*_5_ to form the α-amino acid d-*p*-tolylglycine (**8**).

The results are summarized in Tables 1 and S2. In run 1, (*S*)-amine **1**-*d*_5_ was submitted to the reaction to afford, after the formation of aminonitrile **7**-*d*_5_ and its six cycles of thermal operation, *anti*-l-**7**-*d*_5_ with greater than 99% ee in 56% yield. By contrast, (*R*)-**1**-*d*_5_ induced the production of *anti*-d-**7**-*d*_5_ with greater than 99% ee (run 2). The stereochemical relationships were reproducible as seen in runs 3 and 4. These results are consistent with observed solubility imbalances; that is, by suspending the diastereomeric mixture of *anti*- and *syn*-**7**-*d*_5_ in epimerizing solution (1 M 1,8-diazabicyclo[5.4.0]undec-7-ene (DBU) in methanol), the enrichment of the *anti*-isomer would be induced in the solid phase, then the heating–cooling cycle amplified the solid-phase chirality of the epimerizable α-position. The step-by-step de enhancement was monitored (Figure 3A). We note that an acidic hydrolysis of **7**-*d*_5_ gave, after the removal of isotopically chiral benzhydryl moiety, the corresponding α-amino acid, *p*-tolylglycine (**8**), without decrease of ee (Figure 3B).

Furthermore, as shown in runs 5–7, even though (*S*)-**1**-*d*_5_ with a lower 65–24% ee was utilized for the Strecker reaction, highly stereoimproved l-aminonitrile **7**-*d*_5_ with greater than 99% ee was obtained as a result of the improvement of solid chirality. Because isotopically chiral amine **1**-*d*_5_ with low ee was used, the de of the stereoimproved aminonitrile is not high, but the stereogenic center at the α-position was strictly controlled as the l-configuration. By contrast, (*R*)-amine **1**-*d*_5_ with a low ee (67–20% ee) also induced the production of highly stereoimproved d-aminonitrile **7**-*d*_5_ with a high ee (runs 8–10). When (*S*)-amine **1**-*d*_5_ with a low labeling ratio by mixing with unlabeled **1** was submitted to the Strecker reaction, after the enhancement of chirality, **7**-*d*_5_ including unlabeled **7** with an l configuration was synthesized in the same stereochemical relationship (runs 11 and 12). By symmetry, (*R*)-**1**-*d*_5_ including 50% and 80% of unlabeled **1** induced the formation of oppositely configured d-aminonitrile **7**-*d*_5_ (including **7**) reproducibly (runs 13 and 14).

The following model of amplification in the solid state might be supposed: nearly equimolar amounts of isotopomers dissolve during the heating step to afford a reduced amount of suspended aminonitrile with amplified ee (de), and then recrystallization (deracemization) occurs during the cooling step without a decrease in the amplified ee (de) under a solution-phase epimerization.^36a^ When the amplification cycle started at a *syn*-**7**-*d*_5_-enriched state, the enhancement could be directed to the unfavored isotopomer to afford *syn*-solid **7**-*d*_5_ in a highly diastereoselective manner (>99% de) (Figure S1).

Chiral isotopomers (*S*)- and (*R*)-**1**-*d*_5_ were both asymmetrically synthesized utilizing a single enantiomer of chiral ligand (*R,R,R*)-**4** to exclude the possibility that contamination from **4** induces the direction of the chiral enhancement of aminonitrile **7**-*d*_5_. The isotope chirality is extremely small when the usual asymmetry is compared, as seen in ligand **4**; therefore, even a small amount of contamination might determine the direction of the enhancement. If trace amounts of the chiral contamination from **4** in the added amine **1**-*d*_5_ were responsible for the asymmetric amplification, it would be expected that the resulting aminonitrile **7**-*d*_5_ exhibits the same l- or d-handedness at the α-position, regardless of the isotope chirality of amine **1**-*d*_5_. As confirmed in Tables 1 and S2, l- and d-aminonitrile were obtained reproducibly corresponding to the isotopically chiral amine **1**-*d*_5_, which can exclude the undesired role of chiral compounds originating from **4**. Note that the formation and amplification of unlabeled l- and d-**7** occurred stochastically without the addition of any chiral materials,^32^ and a stereoselective Strecker synthesis has been achieved with the addition of a chiral compound such as amino acids acting as a source and trigger of the amplification of solid-state aminonitrile **7**.^36^

Thus, in the reactions shown in Tables 1 and S2, the hydrogen isotope chirality alone can tip the initial imbalance of the isotopomers in the solid state; then the heating–cooling cycle under epimerization improved the solid-state chirality to give highly diastereomerically enriched and, of course, enantiomerically enriched aminonitrile **7**-*d*_5_.

In the previous report using chirally modified benzhydrylamine, that is, 2-methylbenzhydrylamine,^41^ the resulting diastereomeric aminonitriles form the separate crystals between various aldehydes; thus, resolutions by dynamic crystallization are applicable to afford single diastereomers. The introduction of isotope chirality to the compound-forming conglomerate is the key of the present observation, because an isotopically chiral carbon center cannot be recognized in the crystallization.

When *anti*-**7**-*d*_5_ was dissolved in an epimerizing solution (DBU/methanol), the ee value decreased to achieve below the level of detection by chiral HPLC analysis (Figure S2). Therefore, rather than the energy difference between the diastereomeric isotopomers, the kinetic effect in the crystallization of both isotopic diastereomers might be more rational to explain the observed solubility difference.

**Figure 4:**
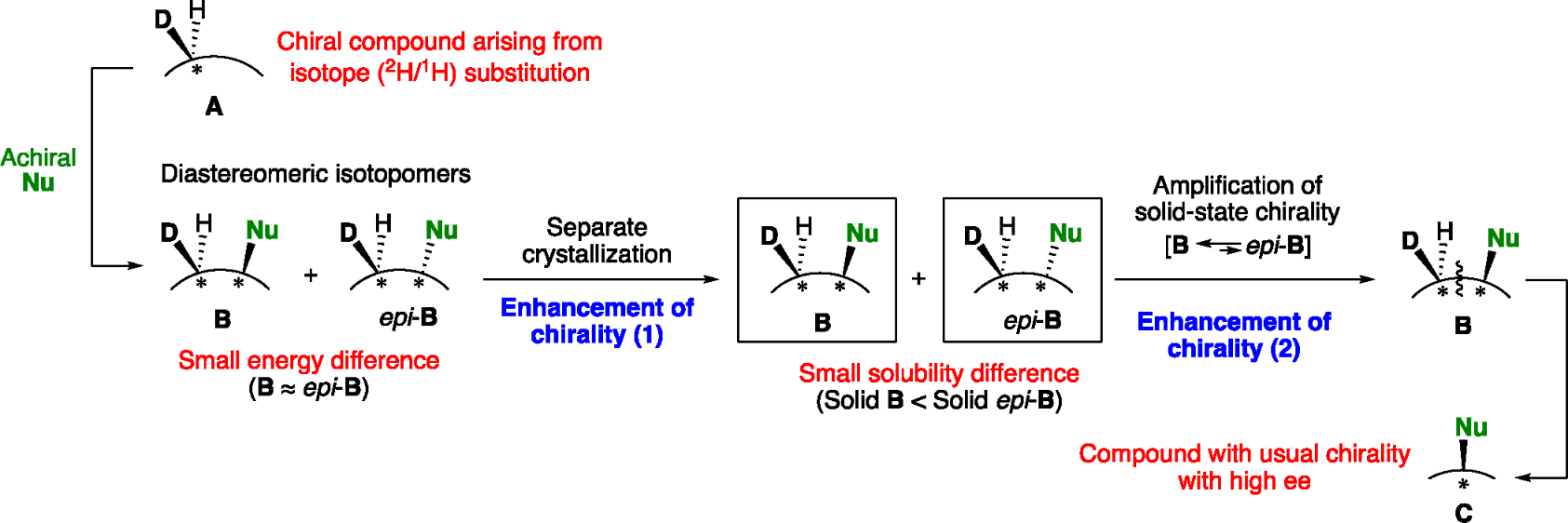
Double enhancement of hydrogen isotope chirality.

A general description of the current double enhancement of hydrogen isotope chirality is outlined in Figure 4. Considering the reaction between chiral compound **A** arising from deuterium substitution and achiral reagent **Nu**, diastereomeric isotopomers **B** and *epi*-**B** should be formed with a new nonisotopic stereogenic center. However, the chiral effect, that is, the diastereoselectivity of **B** and *epi*-**B**, might be negligibly small, and the energy difference between them should be also small. However, when the diastereomers **B** and *epi*-**B** were crystallized separately, the tiny energy difference at the molecular level is accumulated and differentiates the solid-state properties; that is, a solubility difference was induced. Furthermore, by introducing the solution-phase epimerization between **B** and *epi*-**B** at the nonisotopic stereogenic center, solid-state chirality, that is, isotopic de, was amplified to form **B** predominantly. Therefore, after removing labeled substituents, highly enantioenriched organic compound **C** (amino acids in the current work) with the corresponding absolute configuration to that of **A** would be formed via the double enhancement of hydrogen isotope chirality.

## Conclusion

In summary, a highly enantioselective Strecker amino acid synthesis has been achieved by utilizing chiral benzhydrylamine arising from hydrogen isotope (^2^H/^1^H) substitution. For the first time, the solubility difference between diastereomeric isotopomers was demonstrated quantitatively by introducing isotope chirality to the compounds forming the conglomerate. Thus, in the sequence, a tiny energy difference between the diastereomeric isotopomers would be largely integrated to appear as a difference of physical properties between the diastereomeric crystalline solids. Moreover, by introducing solution-phase epimerization, the solid-state chirality of aminonitrile has been significantly enhanced by thermal control to afford the highly stereoimproved α-aminonitrile, which could be hydrolyzed to an α-amino acid with the corresponding absolute configuration of the isotope chirality of the source compound. Therefore, starting from the chiral isotopomer amine, despite its low ee and with a low labeling ratio, the initial isotope chirality was enhanced twice to give the highly enantioenriched α-aminonitrile. Thus, the present observations indicate that an isotopic substitution of achiral compounds is a potential origin and trigger of biological homochirality.

## Experimental Section

### Synthesis of Diastereomeric Mixture of *anti*- and *syn*-Aminonitriles 7-*d*_5_

To a solution of (*R*)-**1**-*d*_5_ (291 mg, 1.55 mmol) in toluene (3.1 mL) and ethanol (1.55 mL) was added *p*-tolualdehyde (**6**) (182 μL, 1.55 mmol) at room temperature. After this solution was stirred for 5 min, the solvents were removed in vacuo. Again, after the addition of toluene (3.1 mL) and ethanol (1.55 mL), solvents were removed in vacuo to afford the crude imine^42^ (455 mg). To a solution of the imine in toluene (4.6 mL) and methanol (4.6 mL) was added HCN (125 μL, 3.1 mmol) at room temperature. After the removal of the solvents and excess HCN in vacuo, aminonitrile **7**-*d*_5_ (489 mg, 1.54 mmol) was obtained as a solid mixture of *anti*-d- and *syn*-l forms.

### Chiral HPLC Analysis of the Supernatant of a Mixture of *anti*- and *syn*-Aminonitrile 7-*d*_5_

A near-equimolar mixture of *anti*-l and *syn*-d aminonitriles **7**-*d*_5_ (70 mg, 0.221 mmol) synthesized from (*S*)-**1**-*d*_5_ was suspended in 2-propanol (4 mL) with stirring. After a precipitation of the solids by stopping the stirring, a part of the clear layer solution was submitted to a chiral HPLC analysis (Daicel Chiralpak IA-3 (4.6 mm × 250 mm), *n*-hexane/2-propanol = 80/20 (v/v), 1.5 mL/min, room temperature, 220 nm, *t*_R_ 7.0 min for *syn*-d-**7**-*d*_5_, 12.9 min for *anti*-l-**7**-*d*_5_) to determine the ratio of diastereomeric isotopomers **7**-*d*_5_.

### Asymmetric Amplification of Aminonitriles 7-*d*_5_^36,41^

A diastereomeric solid mixture of aminonitrile **7**-*d*_5_ (489 mg, 1.54 mmol) was dissolved in dichloromethane and separated into screw-topped vials. After the addition of hexane, the solvents were removed in vacuo with stirring to form the powdered solid **7**-*d*_5_ (140 mg, 0.441 mmol), which was suspended in methanol (1.0 mL). After the mixture was stirred overnight, DBU (0.2 mL) and HCN (36 μL) were added. After a partial (ca. 80–90%) dissolution of suspended solid **7**-*d*_5_ at 45–50 °C, the remaining solid regrew during the gradual cooling to room temperature over 1 h. This thermal cycle was conducted six times to give, as recovered by filtration, *anti*-d-**7**-*d*_5_ (75.3 mg, 0.232 mmol) as a white solid in 53% yield. The ratio of l- and d-**7**-*d*_5_ was determined by HPLC on a chiral stationary phase.

## Supporting Information Available

Methods, synthetic and experimental procedures, characterization of synthesized compounds, supplementary tables and figures (PDF)
