# Supplementary material for: Quantitative Difference in Solubility of Diastereomeric (2H/1H)-Isotopomers
Source: J Am Chem Soc. 2021 Nov 5;143(46):19525–31. doi: 10.1021/jacs.1c09253 (PMC8630799; doi:10.1021/jacs.1c09253)
Supplement: Supplementary file 1 — ja1c09253_si_001.pdf [file ja1c09253_si_001.pdf]

**Quantitative Difference in Solubility of Diastereomeric ($^2H/^1H$)-Isotopomers**

Tsuneomi Kawasaki[a]*, Hiroki Kubo[a], Satoshi Nishiyama[b], Taiki Saijo[a], Rintaro Yokoi[a], Yuji Tokunaga[b]

[a] Department of Applied Chemistry, Tokyo University of Science, Kagurazaka, Shinjuku-ku, Tokyo 162-8601, Japan
[b] Department of Materials Science and Engineering, University of Fukui, Bunkyo, Fukui 910-8507, Japan

*Email: tkawa@rs.tus.ac.jp

## Supporting Information

**Table S1**. Full numerical data of Figure 2B.
**Table S2**. Additional experimental data for stereochemical relationships between the chiral isotopomer benzhydrylamine-$d_5$ (**1**-$d_5$) and resulting α-aminonitrile **7**-$d_5$ (including unlabeled **7**).
**Figure S1.** Enhancement of solid-state chirality to unfavoured *syn*-diastereomeric isotopomers **7**-$d_5$.
**Figure S2.** Solution-phase epimerization of *anti*-**7**-$d_5$.

Experimental methods and the characterization of synthesized compounds.

**Table S1.** Full numerical data for Figure 2B.

| run | amine **1**-$d_5$ (including **1**) | | | batch# of suspension | % ee (config.) of supernatant of **7**-$d_5$ (including **7**)[c] | |
|---|---|---|---|---|---|---|
| | | reaction batch#[a] | % ee | | | average |
| 1 | (*S*)-**1**-$d_5$ | #01$_S$ | 85 | #01$_S$-I | 0.748 (D) | 0.555 (D) |
| 2 | | | | | 0.778 (D) | |
| 3 | | | | | 0.810 (D) | |
| 4 | | | | #01$_S$-II | 0.500 (D) | |
| 5 | | | | | 0.516 (D) | |
| 6 | | | | | 0.504 (D) | |
| 7 | | #02$_S$ | 86 | #02$_S$-I | 0.402 (D) | |
| 8 | | | | | 0.456 (D) | |
| 9 | | | | | 0.480 (D) | |
| 10 | | | | #02$_S$-II | 0.536 (D) | |
| 11 | | | | | 0.534 (D) | |
| 12 | | | | | 0.394 (D) | |
| 13 | (*R*)-**1**-$d_5$ | #03$_R$ | 90 | #03$_R$-I | 0.738 (L) | 0.612 (L) |
| 14 | | | | | 0.518 (L) | |
| 15 | | | | | 0.484 (L) | |
| 16 | | | | #03$_R$-II | 0.504 (L) | |
| 17 | | | | | 0.768 (L) | |
| 18 | | | | | 0.428 (L) | |
| 19 | | #04$_R$ | 93 | #04$_R$-I | 0.700 (L) | |
| 20 | | | | | 0.682 (L) | |
| 21 | | | | | 0.690 (L) | |
| 22 | | | | #04$_R$-II | 0.658 (L) | |
| 23 | | | | | 0.700 (L) | |
| 24 | | | | | 0.468 (L) | |
| 25 | Achiral **1** | –[b] | – | #I | 0.044 (D) | 0.059 (D) |
| 26 | | | | | 0.008 (D) | |
| 27 | | | | | 0.172 (D) | |
| 28 | | | | #II | 0.028 (L) | |
| 29 | | | | | 0.081 (D) | |
| 30 | | | | | 0.074 (D) | |
| 31 | *rac*-**1**-$d_5$ | #05$_{rac}$ | – | #05$_{rac}$-I | 0.004 (D) | 0.009 (L) |
| 32 | | | | | 0.060 (L) | |
| 33 | | | | | 0.028 (D) | |

[a] Identification of **1**-$d_5$ (**1**) synthesized from different reaction batch.
[b] Commercial source was used after distillation.
[c] The ee value was determined by HPLC on a chiral stationary phase.

**Table S2.** Additional experimental data for: Stereochemical relationships between the chiral isotopomer benzhydrylamine-$d_5$ (**1**-$d_5$) and resulting α-aminonitrile **7**-$d_5$ (including unlabeled **7**).[a]

| run | config. of amine **1**-$d_5$ (% ee) [Reaction batch number][b] | unlabeled **1**[b] (%) | aminonitrile **7**-$d_5$ (including **7**) config.[c] (% ee) | aminonitrile **7**-$d_5$ (including **7**) yield[d] (%) |
|---|---|---|---|---|
| 15 | *S* (92) [#12$_S$] | – | L (85) | 50 (31) |
| 16 | *S* (92) [#12$_S$] | – | L (97) | 53 (23) |
| 17 | *S* (89) [#13$_S$] | – | L (>99) | 48 (30) |
| 18 | *S* (89) [#13$_S$] | – | L (>99) | 49 (29) |
| 19 | *S* (89) [#13$_S$] | – | L (>99) | 53 (27) |
| 20 | *S* (89) [#08$_S$] | – | L (98) | 34 |
| 21 | *S* (89) [#08$_S$] | – | L (98) | 42 |
| 22 | *R* (89) [#14$_R$] | – | D (>99) | 53 (32) |
| 23 | *R* (89) [#14$_R$] | – | D (>99) | 52 (22) |
| 24 | *R* (87) [#15$_R$] | – | D (86) | 53 (27) |
| 25 | *R* (87) [#15$_R$] | – | D (95) | 50 (13) |
| 26 | *R* (87) [#15$_R$] | – | D (98) | 53 (14) |
| 27 | *R* (89) [#09$_R$] | – | D (98) | 46 |
| 28 | *R* (89) [#09$_R$] | – | D (98) | 51 |
| 29 | *R* (89) [#09$_R$] | – | D (93) | 48 |
| 30 | *S* (42) [#06$_S$ and #07$_R$] | – | L (99) | 50 (33) |
| 31 | *S* (45) [#10$_S$ and #11$_{rac}$] | – | L (97) | 33 |
| 32 | *S* (14) [#10$_S$ and #11$_{rac}$] | – | L (94) | 33 |
| 33 | *S* (14) [#10$_S$ and #11$_{rac}$] | – | L (74) | 32 |
| 34 | *R* (48) [#04$_R$ and #11$_{rac}$] | – | D (96) | 48 |
| 35 | *R* (31) [#04$_R$ and #11$_{rac}$] | – | D (98) | 36 |
| 36 | *R* (31) [#04$_R$ and #11$_{rac}$] | – | D (25) | N.D. |
| 37 | *R* (21) [#04$_R$ and #11$_{rac}$] | – | D (72) | 23 |
| 38 | *R* (21) [#04$_R$ and #11$_{rac}$] | – | D (72) | 21 |
| 39 | *S* (90) [#10$_S$] | 50 | L (96) | 21 |
| 40 | *S* (90) [#10$_S$] | 80 | L (99) | 15 |
| 41 | *S* (90) [#10$_S$] | 80 | L (99) | 20 |
| 42 | *R* (93) [#04$_R$] | 50 | D (98) | 24 |
| 43 | *R* (93) [#04$_R$] | 80 | D (99) | 26 |
| 44 | *R* (93) [#04$_R$] | 80 | D (99) | 12 |

[a] The molar ratio of **1**-$d_5$ (+**1**) : aldehyde **6** = 1:1, and an excess amount of HCN was used.

[b] Identification of **1**-$d_5$ synthesized from different reaction batch. Labeled amine **1**-$d_5$ with a low ee was prepared by mixing enantioenriched (*S*)-**1**-$d_5$ with (*R*)-**1**-$d_5$ or enantioenriched (*S*/*R*)-**1**-$d_5$ with *rac*-**1**-$d_5$ (reaction batch number #11$_{rac}$) which was prepared from benzonitrile and PhMgBr-$d_5$ and following one-pot $LiAlH_4$ reduction of the resulting iminium salt.

[c] An HPLC analysis using a chiral stationary phase cannot discriminate the isotopic chiral carbon center. Therefore, the ratio of *anti*-L- and *syn*-L-**7**-$d_5$ the same as *anti*-D- and *syn*-D-**7**-$d_5$ could not be determined and the value observed was described as the ee of L- and D-aminonitrile **7**-$d_5$ and **7**.

[d] The chemical yield of solid **7**-$d_5$ by the filtration. The recovered yield of **7**-$d_5$ from the filtrate is indicated in parentheses.

**Figure S1.** Enhancement of solid-state chirality to unfavoured *syn*-diastereomeric isotopomers **7**-$d_5$. The amplification of solid chirality started after the addition of **a,** *syn*-L-**7**-$d_5$ and **b,** L-**7** to near equimolar mixture of *syn*-L- and *anti*-D-**7**-$d_5$.

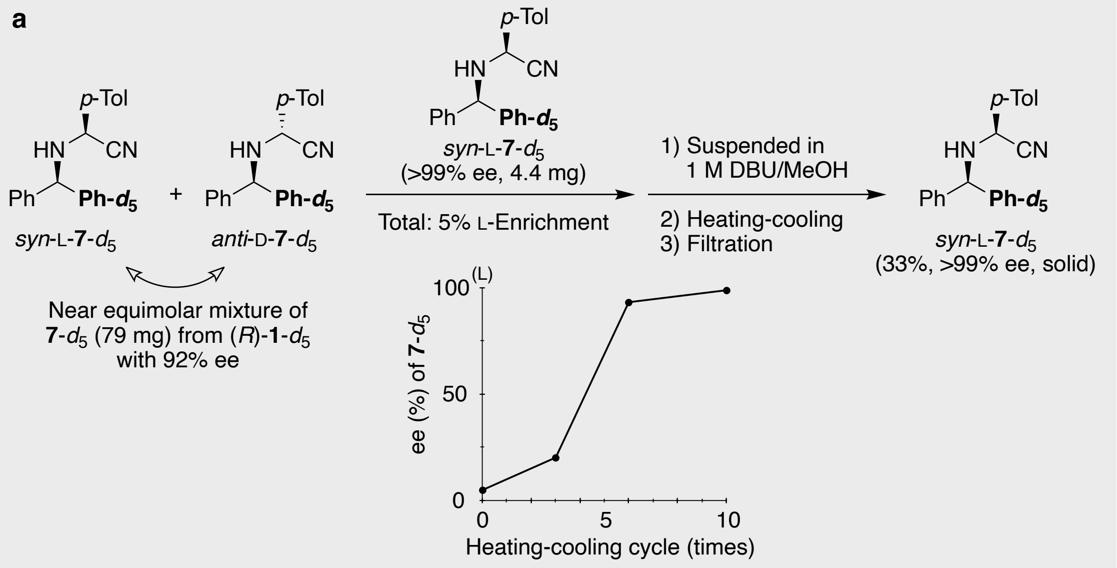


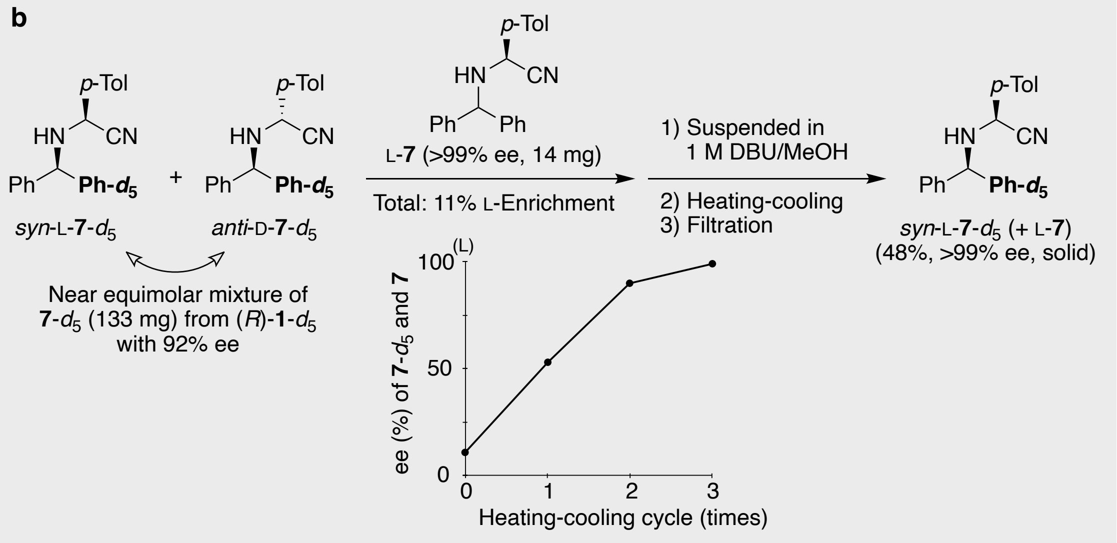

**Figure S2.** Solution-phase epimerization of *anti*-**7**-$d_5$. Aminonitrile *anti*-D-**7**-$d_5$ with 82% ee (11.3 mg) synthesized from (*R*)-**1**-$d_5$ with 89% ee, was dissolved in methanol (3.0 mL) in the presence of DBU (1 μL) and HCN (5 μL) at room temperature. Small amount of HCN was added to prevent the retro-reaction forming imine.[1] The change in ee over time was monitored by using HPLC on a chiral stationary phase. The ee value decreased to 34.8% ee (D) and 0.28% ee (D) after 3 and 18 hours, respectively. Finally, after 65 hours, the ee value achieved to 0.04% ee (D) as an average value of three HPLC measurements, *i.e.*, the ee value is below the level of detection of the current analysis as seen in Table 2B.

Again, the experiment was performed using the enantiomeric aminonitrile, thus, *anti*-L-**7**-$d_5$ with 95% ee (10.8 mg) synthesized from (*S*)-amine **1**-$d_5$ with 65% ee, was dissolved in methanol (3.0 mL) including DBU (1 μL) and HCN (5 μL). The ee value decreased to 31.9% ee (L) and 0.41% ee (L) after 3 and 18 hours, respectively. Finally, after 65 hours, the value achieved to 0.007% ee (L) as an average of three measurements, *i.e.*, the ee value is below the level of detection.

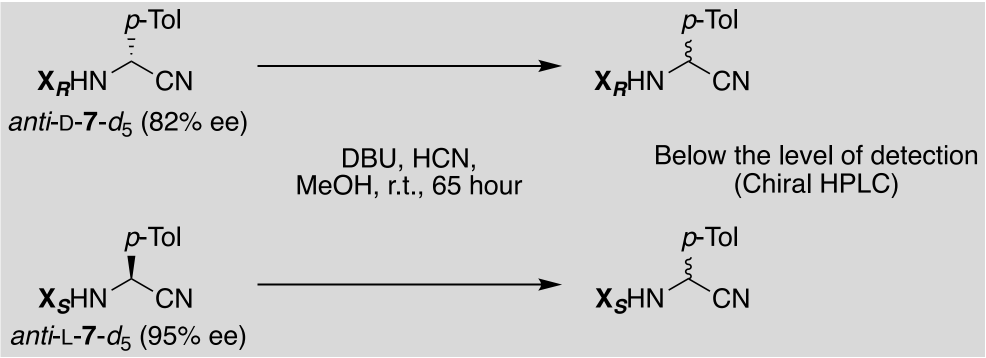

**Experimental methods and the characterization of synthesized compounds.**

**General comments:** *p*-Tolualdehyde (**6**), unlabeled benzhydrylamine (**1**), DBU, methanol and 2-propanol were used after the distillation of commercial sources. Hydrogen cyanide was prepared from $H_2SO_4$ and KCN (or NaCN) in water and isolated by the distillations. Melting points were recorded on an As One ATM-01 apparatus and are uncorrected. $^1H$ NMR and $^{13}C$ NMR spectra were recorded using a JEOL JNM-ECX500II FT NMR system. The chemical sifts δ are given in parts per million (ppm) and the coupling constants J in hertz (Hz). Optical rotations were measured using a JASCO P-2100 digital polarimeters using 5.0 cm cells.

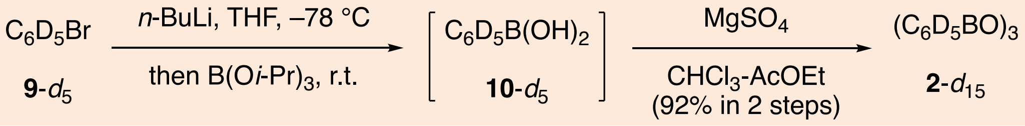


**Tris(phenyl-*d*$_5$)boroxine (2-*d*$_{15}$)**: To a solution of bromobenzene-*d*$_5$ (**9**-*d*$_5$) (1.97 mL, 18.5 mmol) in THF (29.4 mL), was added dropwise a 1.55 M butyllithium in hexane (12.5 mL, 19.4 mmol) for a period of 20 min at –78 °C. After stirring for 2 h, a solution of triisopropyl borate (5.22 mL, 27.8 mmol) in THF (2.94 mL) was added and the mixture was stirred for overnight at room temperature. The reaction was quenched with 20% aqueous HCl (34 mL) at 0 °C and extracted with ether (3 times). The organic layer was washed with water and evaporated in vacuo. The residue was dissolved in a mixed solvent of $CHCl_3$ (32 mL) and ethyl acetate (4.5 mL) and was dried over anhydrous $MgSO_4$. After filtration and concentration, crude solid was purified by washing with hexane to give labeled triphenylboroxine (**2-*d*$_{15}$**) (1.86 g, 5.69 mmol) in 92% yield as a white solid.
**Analytical data**: m.p. 183.0-184.2 °C; FT-IR (ATR) ν 1568, 1465, 1390, 1339, 1303; $^{13}C$ NMR (125 MHz, DMSO-*d*$_6$, 39.52 ppm) 133.7 (m), 133.1 (m), 126.9 (m).

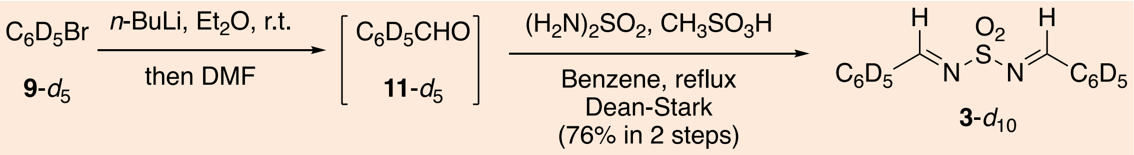


***N,N*'-Bis(phenyl-*d*$_5$-methylidene)sulfamide (3-*d*$_{10}$):** To a solution of **9**-*d*$_5$ (3.29 mL, 30.9 mmol) in ether (50.9 mL), was added dropwise a 1.55 M butyllithium in hexane (20.9 mL, 32.4 mmol) for a period of 20 min at 0 °C. After stirring for 3.5 h at room

temperature, dimethylformamide (DMF: 2.63 mL, 34.0 mmol) was added and the mixture was stirred overnight. The reaction was quenched with 1 M aqueous HCl (17 mL) at 0 °C and extracted with ether (3 times). The organic layer was washed with saturated aqueous $NaHCO_3$ and brine, and then dried over anhydrous $Na_2SO_4$. Evaporation *in vacuo* at 0 °C afforded crude benzaldehyde-$d_5$ (**11**-$d_5$) (3.09 g) and which was dissolved in benzene (32.2 mL). To this solution, sulfamide (1.34 g, 13.9 mmol) and methanesulfonic acid (45 µL, 0.694 mmol) was added. The mixture was refluxed overnight and resulting water was removed using Dean-Stark apparatus. The solid product was collected by the filtration at 0 °C and washed with 2-propanol to give **3**-$d_{10}$ (3.3 g, 11.7 mmol) in 76% yield as a white solid.

**Analytical data for 3-$d_{10}$**: m.p. 88.5-89.5 °C; FT-IR (ATR) ν 1623, 1589, 1562, 1462, 1326, 1142; $^1$H NMR (500 MHz, $CDCl_3$, 0 ppm (TMS)) δ 9.19 (s, 2H); $^{13}$C NMR (125 MHz, DMSO-$d_6$, 39.69 ppm) δ 173.7 (s), 135.0 (m), 132.1 (s), 131.0 (m), 128.9 (m).

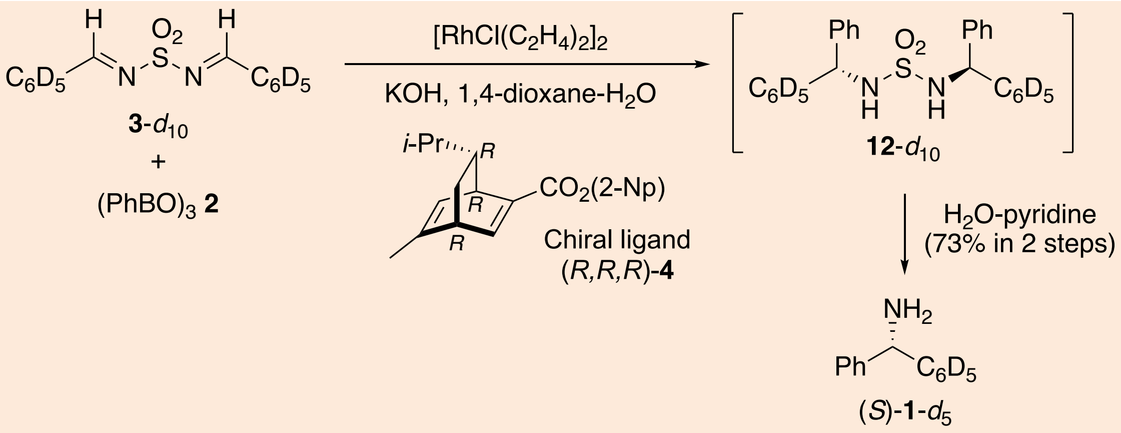


**(*S*)-Phenyl(phenyl-$d_5$)methylamine (1-$d_5$)**:[2] To a solution of (*R,R,R*)-**4** in 1,4-dioxane (1.98 mL), was added chlorobis(ethylene)rhodium (I) dimer (14.5 mg, 37.2 µmol) and was stirred 10 min. To this mixture, was added **3**-$d_{10}$ (350 mg, 1.24 mmol), unlabeled 2,4,6-triphenylboroxine (**2**) (927 mg, 2.98 mmol), 3.1 M aqueous KOH solution (0.16 mL) and 1,4-dioxane (5.94 mL) and was stirred 3 h at 100 °C. After cooling at room temperature, the mixture was passing through silica-gel using ether and concentrated *in vacuo*. Resulting crude product was dissolved in a mixed solvent of 5% $H_2O$ solution in pyridine (54.5 mL) and refluxed overnight. After adding toluene, the volatiles were removed in vacuo. The ethereal solution of the residue was washed with 1 M aqueous HCl. The water layer was neutralized with 6 M aqueous NaOH and was extracted using

ether. The organic layer was dried over anhydrous $Na_2SO_4$ and concentrated in vacuo. The residue was purified with silica-gel column chromatography using a mixed solvent of $CHCl_3$-MeOH (50/1, *v*/*v*) as an eluent to afford (*S*)-**1**-*d*$_5$ (339 mg, 1.80 mmol) in 73% yield as a colourless liquid. The product was used after distillation under reduced pressure.

**Analytical data for (*S*)-1-*d*$_5$**: b.p. 125 °C (0.5 mmHg); d. 1.1986; FT-IR (ATR) ν 3368, 3058, 2843, 1600, 1490, 1450, 894, 698; $^1$H NMR (500 MHz, $CDCl_3$, 0 ppm (TMS)) δ 1.77 (br s, 2H), 5.22 (s, 1H), 7.22 (br t, $J$ = 7.0, 1H), 7.31 (br t, $J$ = 7.5, 2H), 7.37 (br d, $J$ = 7.5, 2H); $^{13}$C-NMR (125 MHz, $CDCl_3$, 77.16 ppm) 145.7, 145.5, 128.5, 128.0 (t, $J$ = 24.4), 127.0, 126.53 (t, $J$ = 23.9), 126.47 (t, $J$ = 24.9) 59.7; HRMS (ESI): Calcd. for $C_{13}H_9D_5N^+$ [M+H$^+$]: 189.1435, Found: 189.1425; $[\alpha]_D^{19}$ 1.40 (neat, ca. 90 % ee, observed α 0.8372); $[\alpha]_{546}^{19}$ 1.68 (neat, ca 90% ee, observed α 1.0063); $[\alpha]_{436}^{19}$ 2.93 (neat, ca. 90% ee, observed α 1.7547); $[\alpha]_{365}^{19}$ 5.31 (neat, ca. 90 % ee, observed α 3.1800).

**Analytical data for (*R*)-1-*d*$_5$:** $[\alpha]_D^{19}$ –1.39 (neat, ca. 90% ee, observed α –0.8327); $[\alpha]_{546}^{19}$ –1.66 (neat, ca. 90% ee, observed α –0.9946); $[\alpha]_{436}^{19}$ –2.93 (neat, ca. 90% ee, observed α –1.7582); $[\alpha]_{365}^{19}$ –5.33 (neat, ca. 90% ee, observed α –3.1966).

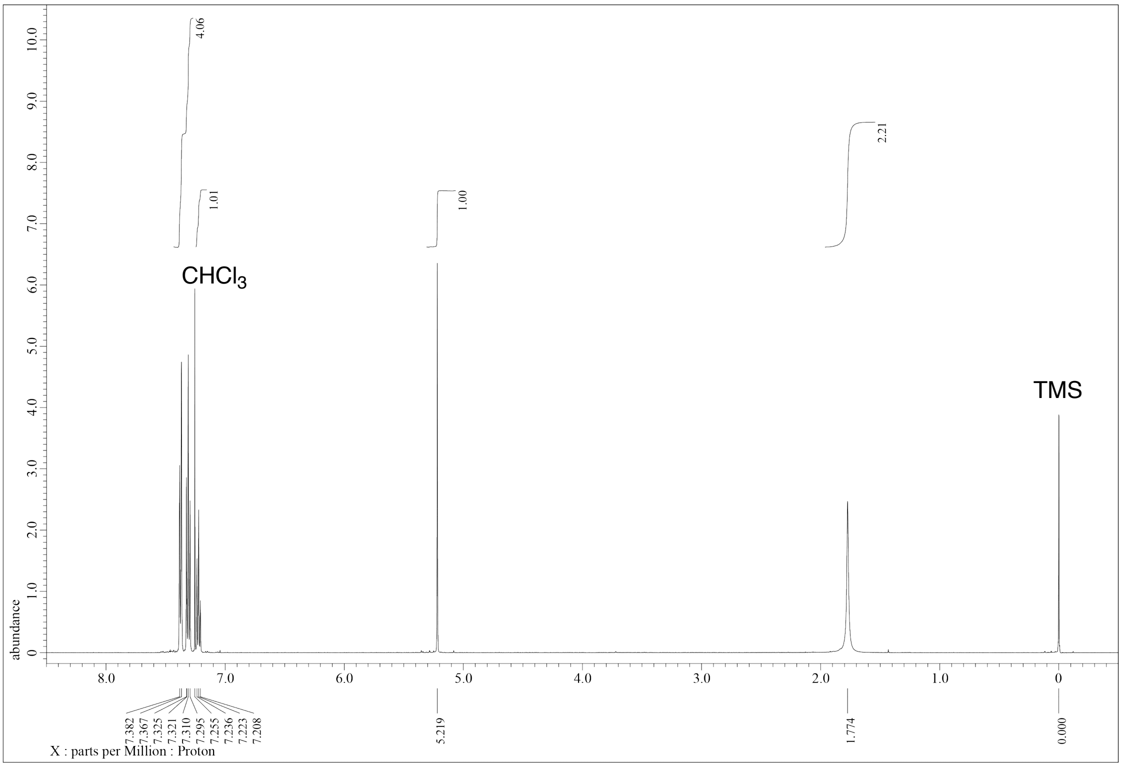


**Figure S3.** $^1$H NMR Spectrum of **1**-*d*$_5$.

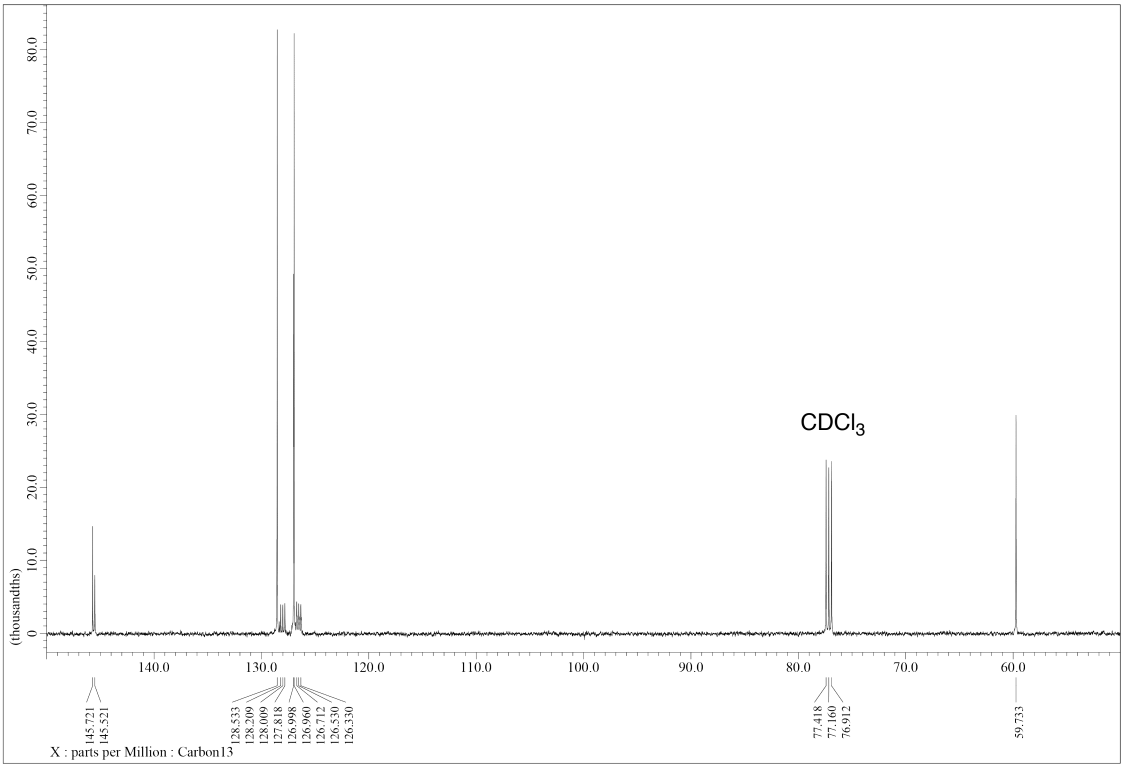


**Figure S4.** $^{13}$C NMR Spectrum of **1**-*d*$_5$.

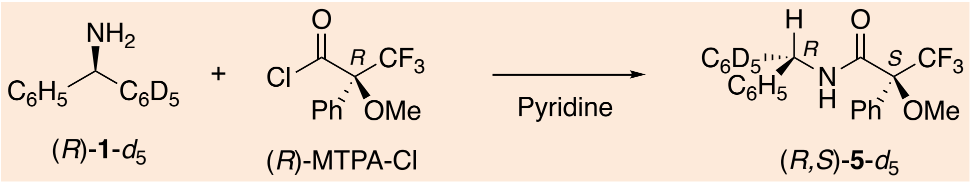


***N*-((*R*)-phenyl(phenyl-*d*$_5$)methyl)-(*S*)-α-methoxy-α-(trifluoromethyl)phenylacetamide (5-*d*$_5$):** To a solution of (*R*)-**1**-*d*$_5$ (12 mg, 0.064 mmol) in pyridine (0.2 mL), was added (*R*)-(–)-α-methoxy-α-(trifluoromethyl)phenylacetyl chloride (17.8 μL, 0.096 mmol, >99% ee). After stirring for 1 h at room temperature, the mixture was diluted with toluene and volatiles were removed under reduced pressure. The residue was purified by passing through silica-gel using $CHCl_3$-methanol (100/1, *v*/*v*) as an eluent followed by preparative thin-layer chromatography ($SiO_2$, hexane / ethyl acetate = 2/1, *v*/*v*) to afford (*R,S*)-**5**-*d*$_5$ (24.6 mg, 0.061 mmol) in 96% yield as a white solid.

**Analytical data for (*R,S*)-5-*d*$_5$:** FT-IR (ATR) ν 3418, 3331, 1696, 1495, 1162, 698; $^1$H NMR (500 MHz, $CDCl_3$, 0 ppm (TMS)) δ 1.25 (br s, 1H), 3.41 (s, 3H), 6.30 (d, *J* = 9.0, 1H), 7.23 (br d, *J* = 7.5, 2H), 7.27–7.42 (m, 6H), 7.48 (br d, *J* = 7.5, 2H); HRMS (ESI):

Calcd. for $C_{23}H_{16}D_5F_3NO_2^+$ [M+H$^+$]: 405.1833, Found: 405.1830.

**Analytical data for (*S,S*)-5-*d*$_5$:** $^1$H-NMR (500 MHz, $CDCl_3$, 0 ppm (TMS)) δ 1.25 (br s, 1H), 3.41 (s, 3H), 6.30 (d, *J* = 9.0, 1H), 7.15 (br d, *J* = 7.5, 2H), 7.28–7.42 (m, 6H), 7.48 (br d, *J* = 7.0, 2H).

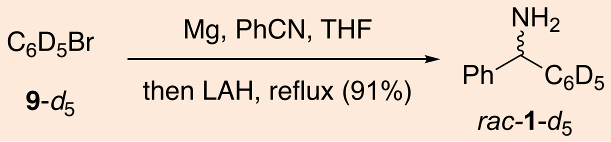


**Phenyl(phenyl-*d*$_5$)methylamine (*rac*-1-*d*$_5$):** To a mixture of magnesium (0.82 g, 33.8 mmol) and catalytic amount of iodine, was added THF (27.8 mL). After disappearance of the colour of iodine, a solution of **9**-*d*$_5$ (3.29 mL, 30.9 mmol, D, 99%) in THF (14.3 mL) was added dropwise for a period of 30 min followed by benzonitrile (3.03 mL, 29.4 mmol). After stirring the mixture overnight under reflux, THF (3.18 mL) and lithium aluminium hydride (1.17 g, 30.9 mmol) was added at 0 °C. After stirring overnight under reflux, the reaction was quenched with water (1.17 mL), 20% aqueous NaOH (1.17 mL) and water (3.51 mL). The mixture was diluted by the addition of THF (24 mL) and the filtrate was concentrated in vacuo. Purification of the residue by silica-gel column chromatography using $CHCl_3$-methanol (100/1, *v*/*v*) as an eluent gave *rac*-**1** *d*$_5$ (5.46 g, 29.0 mmol) in 99% yield, which was used after distillation.

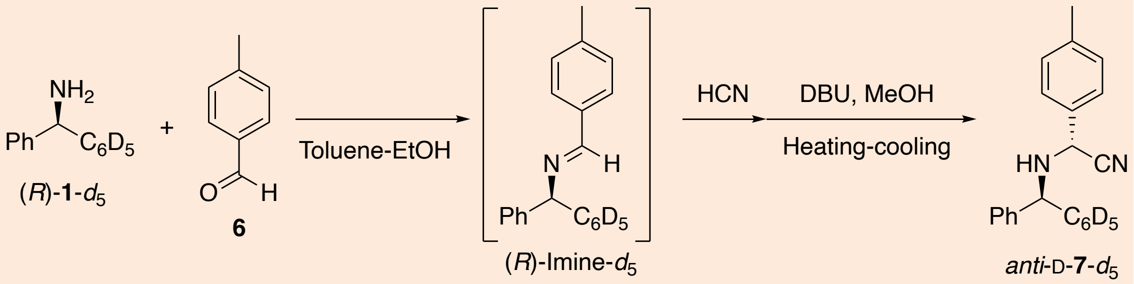


***N*-((*R*)-phenyl(phenyl-*d*$_5$)methyl)-D-α-(*p*-tolyl)glycine nitrile (*anti*-D-8-*d*$_5$) (Table 1, run 22):** To a solution (*R*)-**1**-*d*$_5$ (291 mg, 1.55 mmol) in toluene (3.1 mL) and ethanol (1.55 mL), was added aldehyde **6** (182 μL, 1.55 mmol) at room temperature. After stirring for 5 min, the solvents were removed *in vacuo*. Again, after the addition of toluene (3.1 mL) and ethanol (1.55 mL), solvents were removed *in vacuo* to afford to afford crude (*R*)-imine-*d*$_5$[3] (455 mg). To a solution of crude (*R*)-imine-*d*$_5$ in toluene (4.6 mL) and methanol (4.6 mL), was added hydrogen cyanide (125 μL, 3.1 mmol) at room temperature. After

the removal of the solvents and excess hydrogen cyanide *in vacuo*, resulting solid aminonitrile **7**-*d*$_5$ (489 mg, 1.54 mmol) was dissolved in dichloromethane and separated into the screw vials. After the addition of hexane, the solvents were removed in vacuo with stirring to form powdered solid **7**-*d*$_5$ (140 mg, 0.441 mmol), and which was suspended in methanol (1.0 mL). After stirring the mixture overnight, 1,8-diazabicyclo[5.4.0]undec-7-ene (DBU) (0.2 mL) and hydrogen cyanide (36 μL) were added. After partial dissolution of suspended solid **7**-*d*$_5$ (ca. 80-90%) in the reaction mixture at 45-50 °C, the remaining solid regrew during the gradual cooling to room temperature over a period of 1 hour. This thermal cycle was repeatedly conducted in six times to give, by the filtration, *anti*-D-**7**-*d*$_5$ (75.3 mg, 0.232 mmol) as a white solid in 53% yield. The ratio of L- and D-**7**-*d*$_5$ was determined by HPLC on a chiral stationary phase.

**Analytical data for *anti*-D-7-*d*$_5$:** HPLC analysis: Daicel chiralpak IA-3 (4.6 × 250 mm), hexane/2-propanol=80/20 (*v*/*v*), 1.5 mL/min, room temperature, 220 nm, $t_R$ 7.0 min for D-**7**-*d*$_5$, 12.9 min for L-**7**-*d*$_5$; m.p. (powdered single crystal) 114.5-115.2 °C (D/L = 95.5/4.5, synthesized from (*R*)-**1**-*d*$_5$ with 92% ee, recrystallized from the equimolar solution of *syn*-and *anti*-**7**-*d*$_5$ in $CH_3CN$); $^1H$ NMR (500 MHz, $CDCl_3$, 0 ppm (TMS)) δ 7.45 (br d, *J* = 7.5, 2H), 7.42 (br d, *J* = 7.5, 2H), 7.30 (br t, *J* = 8, 2H), 7.24–7.21 (m, 3H), 5.23 (s, 1H), 4.56 (d, *J* = 12, 1H), 2.37 (s, 3H) 2.10 (d, *J* = 12.5, 1H); $^{13}C$ NMR (125 MHz, $CDCl_3$, 77.16 ppm) δ 142.9 141.1, 139.1, 132.1, 129.8, 128.9, 127.8, 127.23, 127.20, 127.2–129.1 (m), 119.0, 65.6, 52.2, 21.3. HRMS (FAB): Calcd. for $C_{22}H_{16}D_5N_2^+$ [M+H$^+$]: 318.2013, Found: 318.2031.

**Analytical data for *syn*-D-7-*d*$_5$:** m.p. (powdered single crystal) 115.0-116.2 °C (D/L = 1/99, synthesized from (*R*)-**1**-*d*$_5$ with 92% ee, recrystallized from the equimolar solution of *syn*-and *anti*-**7**-*d*$_5$ in $CH_3CN$); $^1H$ NMR (500 MHz, $CDCl_3$, 0 ppm (TMS)) δ 2.11 (br d, *J* = 12.5, 1H), 2.37 (s, 3H), 4.56 (d, *J* = 12.5, 1H), 5.23 (s, 1H), 7.22 (br d, *J* = 8, 2H), 7.29 (br d, *J* = 8, 1H), 7.37 (br t, *J* = 8, 2H), 7.42 (br d, *J* = 8, 2H), 7.56 (br d, *J* = 7.5, 2H). $^{13}C$ NMR (125 MHz, $CDCl_3$, 77.16 ppm) δ 142.7, 141.3, 139.1, 132.1, 129.8, 129.1, 128.0, 127.5, 127.3, 126.6–128.9 (m), 119.0, 65.6, 52.2, 21.3.

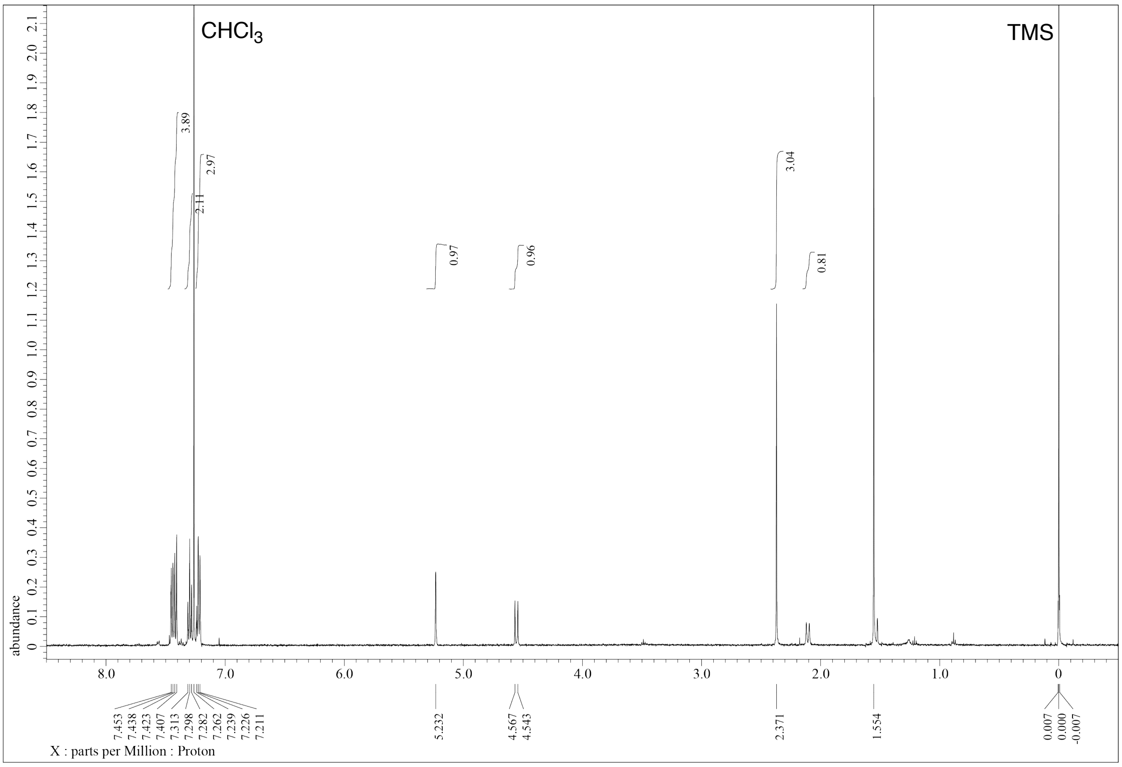


**Figure S5.** $^{1}$H NMR Spectrum of *anti*-**7**-$d_5$.

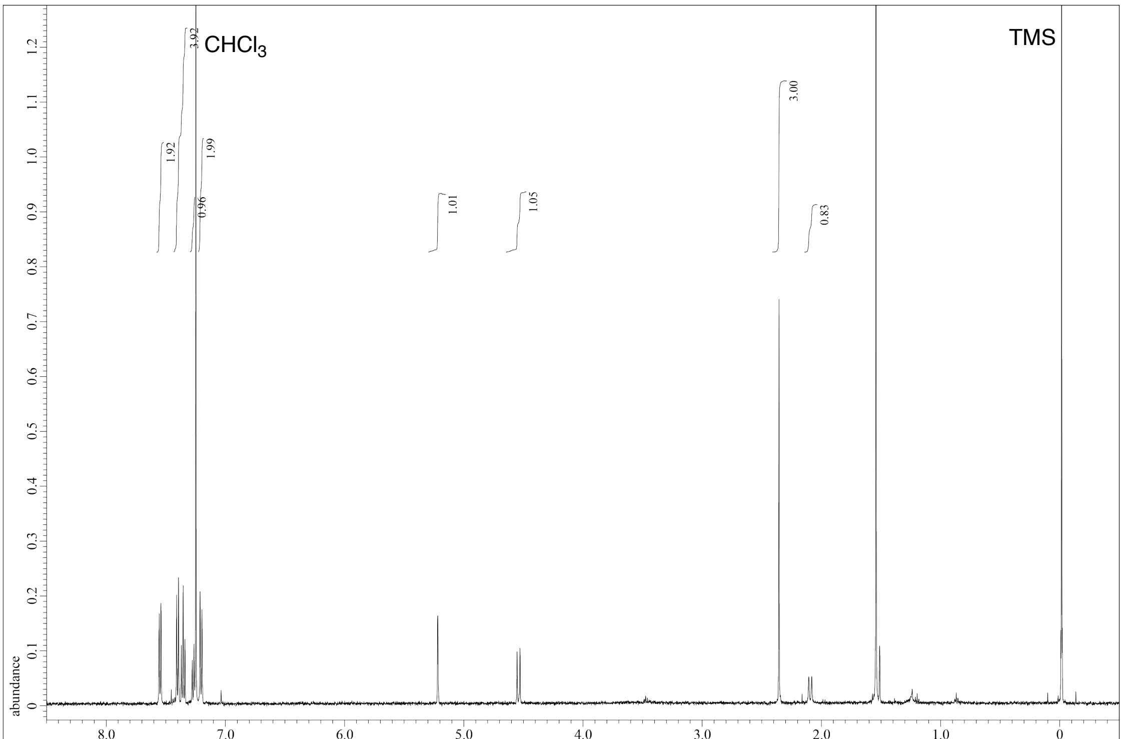


**Figure S6.** $^{1}$H NMR Spectrum of *syn*-**7**-$d_5$.

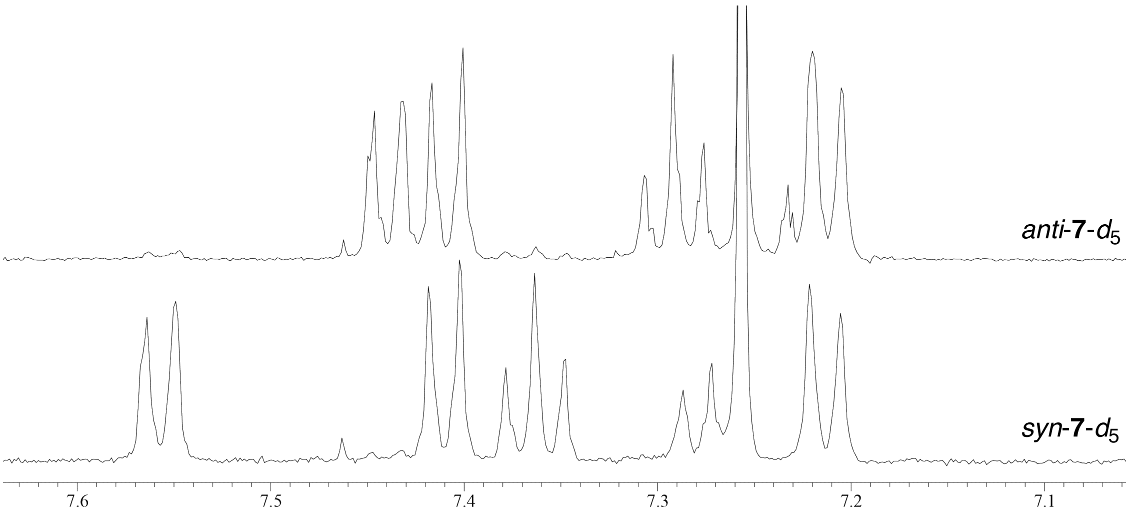


**Figure S7.** Comparison of $^{1}$H NMR spectra between *anti*- and *syn*-**7**-$d_5$.

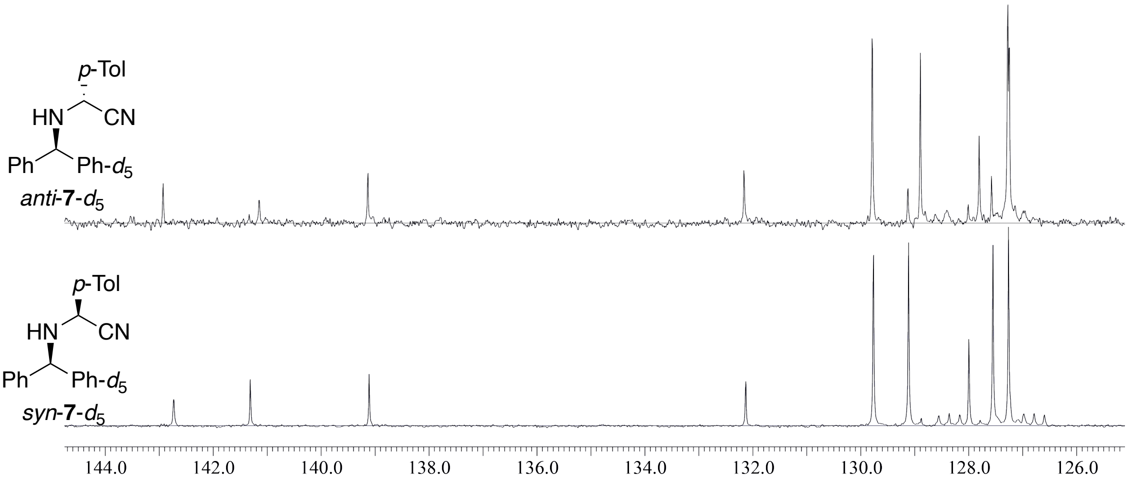


**Figure S8.** Comparison of $^{13}$C NMR spectra between *anti*- and *syn*-**6**-$d_5$.

**Table 2.** HPLC chromatogram of clear layer of the suspension of *syn*-D- and *anti*-L-**7**-$d_5$ formed from (*S*)-benzhydrylamine **1**-$d_5$ (Extended Data Table 1, Run 1: 0.748% D-enrichment).

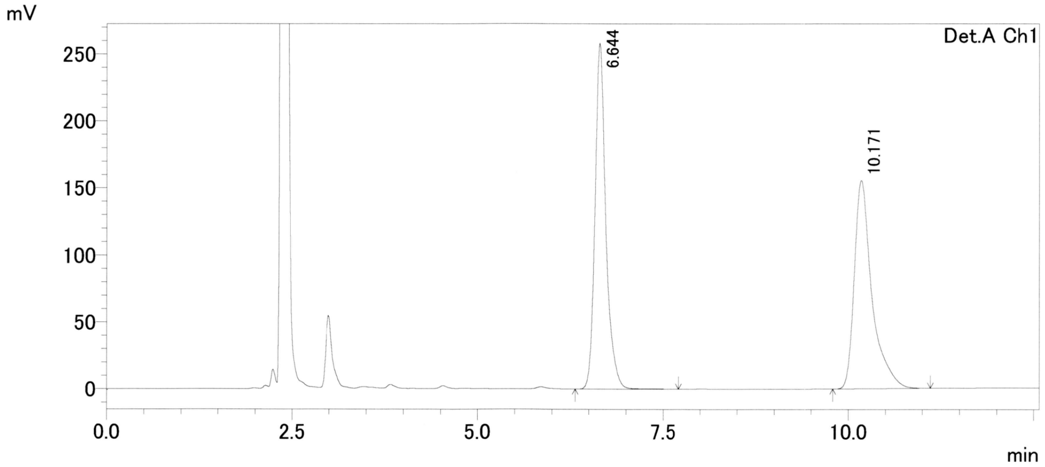


| Compound | $t_R$ [min] | area [µV·sec] | area [%] |
|---|---|---|---|
| *syn*-D-**7**-$d_5$ | 6.644 | 2,613,105 | 50.374 |
| *anti*-L-**7**-$d_5$ | 10.171 | 2,574,317 | 49.626 |

**Table 3.** HPLC chromatogram of clear layer of the suspension of *syn*-L- and *anti*-D-**7**-$d_5$ formed from (*R*)-benzhydrylamine **1**-$d_5$ (Extended Data Table 1, Run 14: 0.518% L-enrichment).

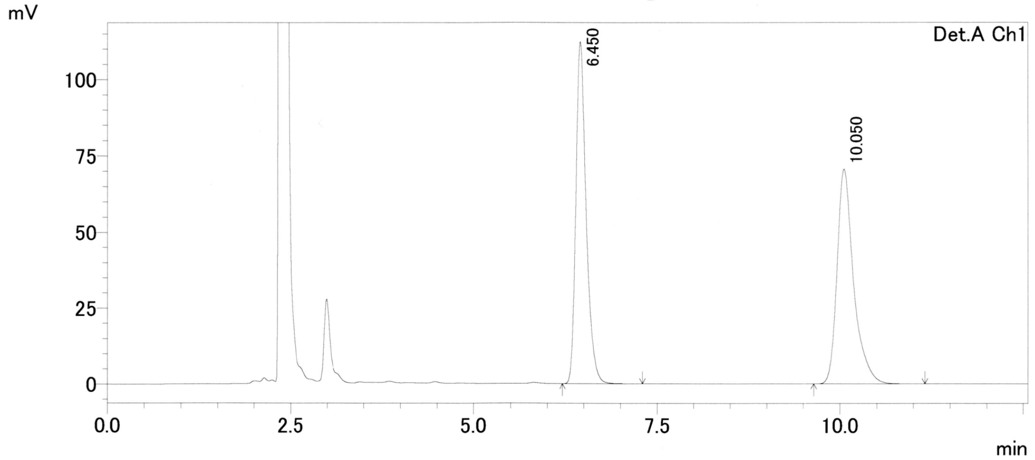


| Compound | $t_R$ [min] | area [µV·sec] | area [%] |
|---|---|---|---|
| *anti*-D-**7**-$d_5$ | 6.450 | 1,095,168 | 49.741 |
| *syn*-L-**7**-$d_5$ | 10.050 | 1,106,582 | 50.259 |

**Table 4.** HPLC chromatogram of clear layer of the suspension of unlabeled L- and D-**7** formed from achiral unlabeled benzhydrylamine **1** (Extended Data Table 1, Run 25: 0.044% D-enrichment).

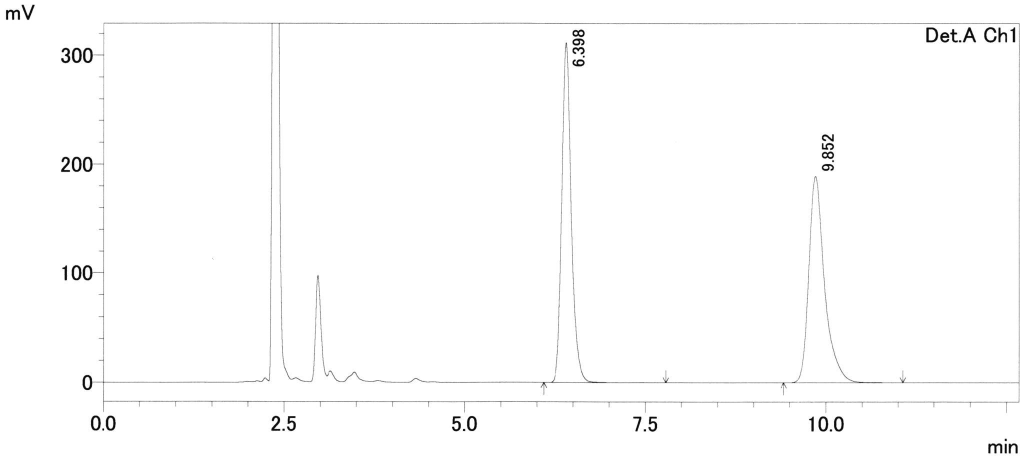


| Compound | $t_R$ [min] | area [μV·sec] | area [%] |
|---|---|---|---|
| D-**7** | 6.398 | 2,862,427 | 50.022 |
| L-**7** | 9.852 | 2,859,957 | 49.978 |

## References


1. Kawasaki, T.; Takamatsu, N.; Aiba, S.; Tokunaga, Y. Spontaneous formation and amplification of an enantioenriched α-amino nitrile: a chiral precursor for Strecker amino acid synthesis. *Chem. Commun.* **2015**, *51*, 14377–14380.
2. Crampton, R.; Woodward, S.; Fox, M. Bis-sulfamyl imines: Potent substrates for asymmetric additions of arylboroxines under rhodium catalysis. *Adv. Synth. Catal.* **2011**, *353*, 903–906.
3. Miyagawa, S., Yoshimura, K., Yamazaki, Y., Takamatsu, N., Kuraishi, T., Aiba, S., Tokunaga, Y. & Kawasaki, T. Asymmetric Strecker reaction arising from the molecular orientation of an achiral imine at the single-crystal face: Enantioenriched L- and D-amino acids. *Angew. Chem. Int. Ed.* **2017**, *56*, 1055–1058.
